# Monitoring guideline adherence in the management of acute coronary syndrome in hospitals: design of a multicentre study

**DOI:** 10.1007/s12471-014-0574-4

**Published:** 2014-07-01

**Authors:** J. Tra, J. Engel, I. van der Wulp, M. C. de Bruijne, C. Wagner

**Affiliations:** 1 Department of Public and Occupational Health - EMGO+Institute/VU University Medical Center, Van der Boechorststraat 7, 1081 BT Amsterdam, the Netherlands; 2 The Netherlands Institute of Health Services Research (NIVEL), Otterstraat 118-124, 3513 CR Utrecht, the Netherlands

**Keywords:** Study protocol, Acute coronary syndrome, Quality indicator, Time-to-treatment, Risk assessment/methods, Acute coronary syndrome/drug therapy

## Abstract

**Background:**

Increasing guideline adherence in the management of acute coronary syndrome (ACS) in hospitals potentially reduces heart failure and mortality. Therefore, an expert panel identified three guideline recommendations as the most important aims for improvement in ACS care, i.e. timely invasive treatment, use of risk scoring instruments and prescription of secondary prevention medication at discharge.

**Aims:**

This study aims to evaluate in-hospital guideline adherence in the care of patients diagnosed with ACS and to identify associated factors.

**Methods:**

The study has a cross-sectional design. Data are collected in 13 hospitals in the Netherlands by means of retrospective chart review of patients discharged in 2012 with a diagnosis of ACS. The primary outcomes will be the percentages of patients receiving timely invasive treatment, with a documented cardiac risk score, and with a prescription of the guideline-recommended discharge medication. In addition, factors associated with guideline adherence will be studied using generalised linear (mixed) models.

**Discussion:**

This study explores guideline adherence in Dutch hospitals in the management of patients diagnosed with ACS, using a data source universally available in hospitals. The results of this study can be informative for professionals involved in ACS care as they facilitate targeted improvement efforts.

## Background

Patients diagnosed with an acute coronary syndrome (ACS) have a high risk of dying from their condition. Mortality rates differ for the three clinical manifestations of ACS: ST-segment elevation myocardial infarction (STEMI), non-ST-segment elevation myocardial infarction (NSTEMI) and unstable angina (UA) [1]. The symptoms of ACS are usually caused by the same pathophysiological mechanism, i.e. coronary stenosis. However, the differences in severity of coronary stenosis and mortality have led to differences in the management of ACS [2, 3].

Improved management strategies for patients diagnosed with ACS have led to a decrease in mortality rates in the past years [4–6]. For patients with STEMI the strategy progressed from acute pharmacological intervention (thrombolysis) to immediate percutaneous coronary intervention (PCI) [7]. In the management of NSTEMI and UA patients, risk scoring instruments were developed and implemented to estimate patients’ future risk of major adverse cardiac events in order to weigh the risks and benefits of invasive treatment [8]. Independent of the type of ACS, prescribing secondary prevention medication further reduces morbidity and prevents additional episodes of ACS [9]. Using the aforementioned strategies increases patients’ chances of survival [10, 11] and these strategies are therefore incorporated in international cardiology guidelines [12, 13].

However, previous studies reported that not all patients are treated according to these guideline-recommended strategies [14, 15]. For example, patients with higher age, female sex, prior heart failure, renal insufficiency or coronary artery bypass graft (CABG) surgery during admission were less likely to receive guideline-recommended discharge medication [16]. Also, variation in guideline adherence between hospitals has been reported [10]. To identify room for improvement in the management of ACS, it is imperative to monitor guideline adherence and to identify associated factors.

The objective of this study is therefore to determine the degree of ACS guideline adherence in Dutch hospitals. A Dutch expert panel identified timely invasive treatment, use of cardiac risk scoring instruments and prescribing guideline-recommended discharge medication as the most important aims for improvement in ACS care. A secondary objective of this study is to explore patient and hospital characteristics associated with guideline adherence. In the present paper the design of the study will be outlined.

## Research questions

To what degree are:

- patients diagnosed with STEMI treated with PCI within 90 min of first (para)medical contact?
- cardiac risk scoring instruments used in the management of patients diagnosed with NSTEMI/UA?
- the recommended medicines for secondary prevention prescribed to patients diagnosed with ACS at discharge from the hospital?

Additionally, what patient and hospital characteristics are associated with guideline adherence?

## Methods/design

### Design

The study has a cross-sectional design.

### Setting

In the Netherlands 30 out of the 91 hospitals offer PCI, of which 16 also provide CABG surgery.

The three guideline recommendations monitored in the present study were identified from the European Society of Cardiology guidelines by an expert panel consisting of cardiologists, an emergency department medical resident, an intensive care/cardiac care nurse and health care scientists. Adherence to these three recommendations is measured over 2012, the last year of a national quality improvement program. The program aims to decrease in-hospital mortality caused by ten high-risk patient safety threats [17], including ACS.

### Selection of hospitals

The study is being conducted in 13 hospitals, selected by means of a multi-stage random sampling procedure. Initially six PCI-capable and six non-PCI-capable hospitals with a cardiology department were randomly selected from a pool of 40 randomly selected hospitals. Three PCI-capable hospitals declined participation, for which three additional PCI-capable hospitals were selected. Because the number of STEMI patients was relatively small, an additional PCI-capable hospital was selected. The hospitals are located in 7 of the 12 Dutch provinces, with bed capacities ranging between 200 and 1200 beds (Table 1).

**Table 1:** Characteristics of hospitals included in the study

Hospital ID	1	2	3	4	5	6	7	8	9	10	11	12	13
Type	Gen	Gen	Gen	Teach	Teach	Gen	Teach	Teach	Teach	Acad	Teach	Teach	Acad
Bed capacity	200–400	200–400	200–400	400–600	400–600	800–1000	600–800	600–800	600–800	600–800	1000–1200	800–1000	800–1000
PCI	No	No	No	No	No	No	Yes	Yes	Yes	Yes	Yes	Yes	Yes
CABG	No	No	No	No	No	No	No	No	Yes	Yes	Yes	Yes	Yes

Due to privacy reasons, bed capacity is categorised and the province per hospital is not included

*Gen* general hospital; *teach* tertiary teaching hospital; *Acad* academic hospital; *PCI* percutaneous coronary intervention; *CABG* coronary artery bypass graft surgery

### Data collection

**Fig. 1:**
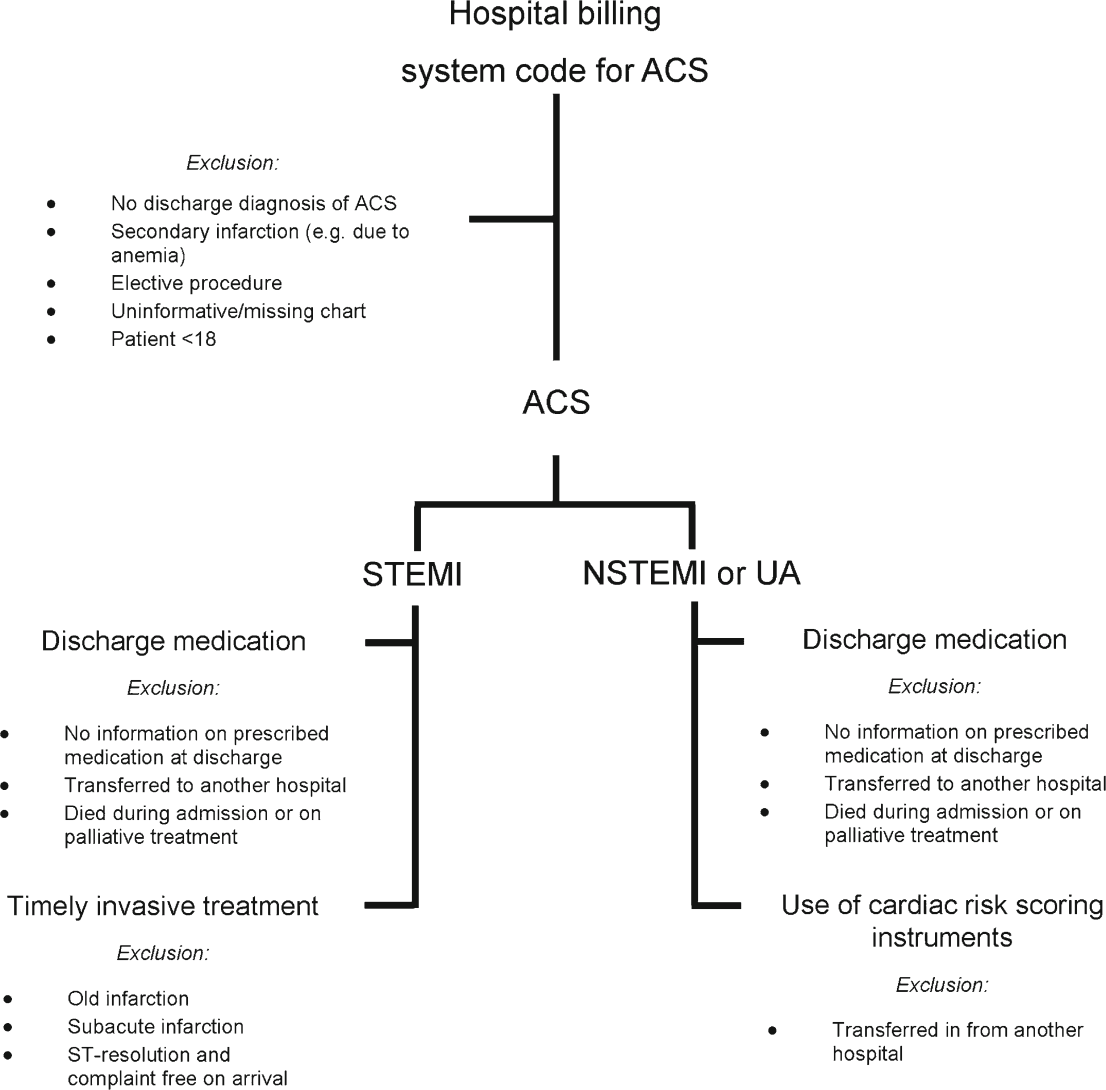
Flow chart of the selection of patient charts. *ACS* acute coronary syndrome; *STEMI* ST-segment elevation myocardial infarction; *NSTEMI* non-ST-segment elevation myocardial infarction; *UA* unstable angina

The data are collected by means of retrospective chart review of electronic and/or paper-based medical, nursing and catheterisation laboratory charts of patients discharged between January 1^st^ and December 31^st^ 2012. Monthly, potential study charts are selected from the hospital billing system using diagnosis-treatment combination codes. Charts of patients discharged with a confirmed diagnosis of ACS (indicated in the discharge letter) are considered for inclusion (Fig. 1). When the discharge diagnosis is unclear, the chart is discussed with a cardiologist or other attending physician working in the field of cardiology. Charts of patients without a discharge diagnosis of ACS, a secondary ACS (e.g. due to anaemia), elective procedures, missing or uninformative charts, and charts of patients under the age of 18 years are excluded from the study. Moreover, additional exclusion criteria were defined for each process indicator separately. For timely invasive treatment, charts of STEMI patients not going for acute PCI are excluded. For use of risk scoring instruments, charts of patients transferred from another hospital are excluded. For discharge medication, charts of patients who were transferred to another hospital, patients who died during their admission or received palliative treatment are excluded.

If the monthly number of charts exceeds the screening capacity, screening of the charts is performed in chronological order of discharge representing STEMI and NSTEMI/UA equally, and terminated when the chart abstractors are practically unable to screen additional charts.

In two hospitals the pre-selection procedure based on the hospital billing system is not possible. Therefore in one hospital the pre-selection of charts is performed by requesting all charts of patients with a suspected or confirmed diagnosis of ACS through the cardiology department’s secretariat. In the other hospital, local hospital regulations require that patients with a suspected ACS are informed about the study and asked to give informed consent before their chart can be considered for inclusion. Due to the declined invitations and deviations in inclusion procedures, data collection in 5 hospitals will comprise 9 or 10 months instead of 12 months.

### Study outcomes

The study has three main outcome measures. First, the percentage of STEMI patients in which the PCI procedure started within 90 minutes from first (para)medical contact. Second, the percentage of NSTEMI or UA patients where use of a validated risk scoring instrument was documented. Finally, the percentage of ACS patients with a prescription of the recommended discharge medication, documentation of a contraindication or other reason for not receiving the recommended medication. Additionally, patient and hospital characteristics associated with guideline adherence will be identified.

### Recorded variables

From all charts, the following information is abstracted: demographic and clinical information including gender, age, cardiac history, risk factors, biomarker values, electrocardiogram findings, resuscitation, heart failure, cardiogenic shock on arrival and month of discharge (Table 2).

**Table 2:** Information recorded for all ACS patients

**General information**	**Discharge medication (yes/no)**
Gender	Acetylsalicylic acid
Date of birth	Thienopyridine
Admission date and time	Statin
Symptoms	Beta blocker
Discharge date	Angiotensin-converting enzyme inhibitor
Discharge status (discharged, deceased, unknown)	
	**Contraindications (yes/no)**
**History of cardiac disease (yes/no)**	*Acetylsalicylic acid*
Coronary vascular disease	Coagulation defect
Peripheral vascular disease	Active peptic ulcer (ulcus pepticum)
(Unstable) angina pectoris	Stroke (bleeding)
Acute myocardial infarction	Liver failure
Coronary artery bypass graft surgery, year: ______	Kidney failure
Percutaneous coronary intervention, year: ______	Allergy/oversensitivity
Intervention/acute myocardial infarction <6 months	Treatment with anticoagulant medication
	G6PD-deficiency
**Risk factors (yes/no)**	Other:
Diabetes mellitus	*Thienopyridine*
Hypertension	Transient ischemic attack/cerebrovascular accident
Kidney failure	Active peptic ulcer (ulcus pepticum)
Chronic heart failure	Liver failure
Positive family history	Pathological bleeding (from ulcus pepticum or intracranial bleeding)
Smoker	
Previous smoker	Other:
Elevated cholesterol levels (statin use in history, hyperlipidaemia, hypercholesterolaemia)	*Statin*
Obesity (body mass index >30 kg/m2)	Liver function impairment
	Renal impairment
Coronary stenosis >50 % (in history)	Other:
Age >70 year	*Beta blocker*
Male sex	Sick-sinus syndrome
Aspirin use (<7 days)	2nd and 3rd degree AV block (ECG)
	Hypotension
**Vital functions**	Cardiogenic shock
Cardiogenic shock (yes/no)	Sinus bradycardia
Heart failure (yes/no)	Unstable or untreated heart failure
Resuscitation (yes/no)	Pheochromocytoma
Blood pressure on arrival (mmHg)	Bronchial asthma (anamnesis)
Heart rate (beats per minute)	Severe peripheral circulation defects
Electrocardiogram date and time	Metabolic acidosis
Electrocardiogram interpretation	Pulmonary hypertension
Biomarker values (troponin, creatinin kinase (CK), creatinin kinase-muscle/brain (CK-MB), creatinin)	Kidney failure
	Liver failure
**Cardiac rehabilitation (yes/no)**	Myocardial infarction with heart frequency <45, P-Q >0.24, systolic blood pressure <100
Enlistment for cardiac rehabilitation	Other:
	*Angiotensin-converting enzyme inhibitor*
	Kidney failure
	Other:

*ACS* acute coronary syndrome

In addition, for the timely invasive treatment indicator, the following variables are recorded: routing of the patient, type of first (para)medical contact, place of first electrocardiogram, type of treatment, and the dates and times of first (para)medical contact, first (ambulance/general practitioner) electrocardiogram and sheath insertion (start of PCI) (Table 3).

**Table 3:** Additional recorded variables for STEMI patients

**General information**
Routing out-of-hospital
Type of treatment (pharmacological, acute percutaneous coronary intervention, non-acute percutaneous coronary intervention, coronary artery bypass graft surgery)
Discipline of first (para)medical contact
Discipline of first electrocardiogram
Number of diseased vessels
Location of stenoses
**Time variables**
Symptom onset
First (para)medical contact
First electrocardiogram
Sheath insertion
First balloon inflation or thrombus aspiration

*STEMI* ST-segment elevation myocardial infarction

To evaluate cardiac risk score adherence, application of a validated risk scoring instrument (e.g. GRACE [18, 19], TIMI [20], FRISC [21], HEART [22] and PURSUIT [8]), type of instrument, risk score outcome, date of application, and type of treatment are recorded (Table 4).

**Table 4:** Additional recorded variables for NSTEMI and UA patients

**General information**
Routing in-hospital
Catheterisation (yes/no)
Type of treatment (pharmacological, percutaneous coronary intervention, coronary artery bypass graft, unknown, other)
**Risk score**
Use of validated risk score (yes/no)
Date of application
Type of instrument(s)
Risk score outcome
Risk score outcome classification
Additional diagnostics

*NSTEMI* Non-ST-segment elevation myocardial infarction; *UA* unstable angina

Finally, for discharge medication, prescription of acetylsalicylic acid, thienopyridine, statin, beta blocker and angiotensin-converting enzyme (ACE) inhibitor and contraindications or other reasons for not prescribing all or some of the medication are recorded (Table 2). Contraindications were derived from an annually updated database containing information about all medication registered in the Netherlands [23].

### Abstraction of data

All data are collected on standard case report forms. Variables are defined in codebooks. Two researchers (JT & JE) developed the codebooks and case report forms based on the European Society of Cardiology guidelines. The case report forms were discussed within the research group, tested in two pilot measurements and adjusted accordingly. The data are collected by six chart abstractors who were introduced to the subject of ACS and instructed in the chart review procedures by JT and JE. Chart reviews were supervised until the quality of the chart reviews was satisfactory. The data are entered into a database using a data entry program with fixed entry fields (BLAISE version 4.7, Statistics Netherlands) and compared with the original case report form by a second researcher.

To ensure reliability of the data and to assess the quality of the codebook, a sample of charts (5–10 %) is independently screened again by one of the five other chart abstractors. The two case report forms are compared, and differences are discussed until consensus is reached. If necessary, changes are made in the original case report form. The reliability between the chart abstractors will be calculated by means of the percentage of agreement for each variable.

### Statistical analyses

#### Missing data

Missing data patterns will be analysed by means of missing value analyses. Depending on the pattern [24], missing values will be imputed by means of a single imputation (missing completely at random) or multiple imputation procedure (missing at random) [25].

#### Descriptive statistics

The degree of adherence to the three process indicators will be presented by descriptive statistics. Associations of patient and hospital characteristics with guideline adherence are studied in separate analyses.

#### Timely invasive treatment

The time to PCI in minutes will be entered as a continuous dependent variable in a generalised linear model taking into account its distribution, as time variables are generally not normally distributed. In univariate analyses, associations of the independent variables, i.e. patient and admission characteristics, are studied. To account for clustering of patient data within hospitals, the variable ‘hospital’ and its significant interactions with any other of the predictor variables will be entered as a covariate in all univariate models [26]. This is because the hospital sample size (7 PCI-capable hospitals) is considered small for multilevel regression analysis [27]. All variables and interactions significantly (*p* ≤ 0.05) associated with the time to PCI will be included in the multiple generalised linear model. Furthermore, to minimise the probability of making a type II error, all non-significant variables from the univariate models will be added to the multiple generalised linear model one by one. Significant variables (*p* ≤ 0.05) will be added to the final model.

#### Use of risk scoring instruments

Associations of independent variables with the use of cardiac risk scoring instruments will be studied by means of a generalised linear mixed model (GLMM). In the analysis the binary dependent variable will be the use of a validated risk score instrument. Independent variables will be patient characteristics, hospital characteristics and month of discharge. To account for clustering of the data, the model will comprise random effects for hospitals. First, independent variables will be tested separately correcting for the random hospital effects. Second, all independent variables with a significance level below *p* ≤ 0.15 will be selected. Next, pairs of selected independent variables will be tested jointly.

Last, all significant (*p* ≤ 0.05) variables from the previous steps will be included in the final multivariable model. This final step also comprises a cautious consideration of significant (*p* ≤ 0.05) interaction terms.

#### Discharge medication

Associations of independent variables with the prescription of the recommended discharge medication will be studied by means of GLMM. In these analyses, prescription of the five guideline-recommended medicines or documentation of contraindications (yes/no) will be the binary dependent variable. The effects of the independent variables including patient, hospital and discharge characteristics will be tested in univariate analyses. All variables with a significant association (*p* ≤ 0.05) with the dependent variable will be included in a multivariable model. To account for the effects of collinearity, all variables not significantly related to prescription of the recommended discharge medication in the univariate models will be added to the multivariable generalised linear mixed model one by one. Interactions will be tested and added to the multivariable model in case of a significant effect. In all models, hospital will be entered as a random effect variable to account for clustering of the data. As not all medicines are indicated for all patients with ACS according to the European Society of Cardiology guidelines (e.g. ACE-inhibitors are recommended for all patients with ACS, but only indicated for those patients with a reduced cardiac function), additional models will be created to analyse the effects of patient and hospital characteristics on the prescription of ≤3 and ≥4 medicines or documentation of a contraindication.

#### Software

The data will be analysed in IBM SPSS Statistics (version 20 for Windows) and R (version 3.0.0 for Windows).

## Ethical approval and confidentiality

The study protocol was approved by the medical ethics review committee of the VU University Medical Center. To protect patients’ and hospitals’ privacy, they are assigned a unique observation code. All data are stored on a password protected network server of the VU University Medical Center, to which only the participating researchers have access. All chart abstractors signed a confidentiality agreement and the study was registered with the Dutch Data Protection Agency.

## Discussion

This paper describes the design of a study of the quality of Dutch ACS care by evaluating the degree to which hospitals adhere to three key quality indicators from (inter)national guidelines and by exploring factors associated with guideline adherence.

Previous North American studies that monitored guideline adherence have successfully identified associated factors [10, 16, 28], after which targeted quality improvement efforts could be applied. These efforts increased the likelihood that patients were treated on time with PCI [29], risk scores were documented [30] and the recommended discharge medication was prescribed [31]. Therefore the monitoring of guideline adherence as the foundation for targeted quality improvement efforts seems promising.

The three guideline recommendations evaluated in this study were selected from the European Society of Cardiology guidelines [12, 13], but are also included in other (inter)national guidelines [32–34]. The methods used in this study can be applied to evaluate the process of ACS care in other countries, especially in countries where large, national registries of guideline adherence are lacking.

### Potential limitations

In designing the study, several limitations have to be taken into account. First, the documented information in the charts and variability between the chart abstractors may affect the reliability of the data. This will be reduced by using standardised case report forms, a codebook and by interim reliability checks of the data. Second, using the diagnosis in the discharge letter as inclusion criterion may not be as reliable as applying our own diagnostic criteria. However, it was considered important to take into account the interpretation of the treating physician at the time of hospitalisation of the patient. Third, the presence of researchers on site, and quarterly feedback from the national quality improvement program might influence hospitals’ performance on the outcomes. However, in a report on the evaluation of the quality improvement program the effect of this national intervention was limited [35]. Finally, the selection of hospitals and patients could not be performed completely randomly due to practical limitations. However, the hospitals included in this study were geographically spread over the country, thereby limiting the influence of potential regional variation in guideline adherence. Additionally the outcomes of this study are corrected for the influence of individual hospitals in the statistical models.

## Conclusion

Evidence-based guidelines are of vital importance in safely and effectively treating patients diagnosed with ACS. The results of this study will provide insight into the degree of guideline adherence in Dutch hospitals for the management of patients with ACS and identify room for further improvement. Furthermore, patient and hospital characteristics associated with guideline adherence will be identified, which may facilitate targeted improvement strategies.
